# Choosing Cell Fate Through a Dynamic Cell Cycle

**DOI:** 10.1007/s40778-015-0018-0

**Published:** 2015-07-01

**Authors:** Xinyue Chen, Amaleah Hartman, Shangqin Guo

**Affiliations:** Department of Cell Biology, Yale Stem Cell Center, Yale University School of Medicine, New Haven, CT 06520 USA

**Keywords:** Cell cycle, Cell fate, Reprogramming, Hematopoietic progenitors, Pluripotency

## Abstract

A close relationship between proliferation and cell fate specification has been well documented in many developmental systems. In addition to the gradual cell fate changes accompanying normal development and tissue homeostasis, it is now commonly appreciated that cell fate could also undergo drastic changes, as illustrated by the induction of pluripotency from many differentiated somatic cell types during the process of Yamanaka reprogramming. Strikingly, the drastic cell fate change induced by Yamanaka factors (Oct4, Sox2, Klf4, and c-Myc) is preceded by extensive cell cycle acceleration. Prompted by our recent discovery that progression toward pluripotency from rare somatic cells could bypass the stochastic phase of reprogramming and that a key feature of these somatic cells is an ultrafast cell cycle (~8 h/cycle), we assess whether cell cycle dynamics could provide a general framework for controlling cell fate. Several potential mechanisms on how cell cycle dynamics may impact cell fate determination by regulating chromatin, key transcription factor concentration, or their interactions are discussed. Specific challenges and implications for studying and manipulating cell fate are considered.

## Introduction

The ability to faithfully maintain cell fate or cell identity is critical for the function of highly specialized cell types in multicellular organisms. At the same time, embryonic development and tissue homeostasis demand that cells adopt a variety of different cell fates. Cell fate refers to a specific gene expression program that is relatively stable and reflective of cellular functions. The need to maintain cell fate stability and faithfulness thus fundamentally conflicts with the acquisition of new cell fates. How do cells solve this conflict and produce two alternative outcomes? Watson-Crick base pairing provides an ultimate mechanism for replicative faithfulness, which forms the basis of the key aspects of cell fate maintenance mechanisms [1, 2]. These mechanisms also include the maintenance DNA methylation system [3] and the histone post-translational modification system [4, 5]. In addition, many transcription factors are known to associate with mitotic chromatins, marking specific genetic loci for transcription activation after mitosis in a process termed “mitotic bookmarking” [6]. In contrast to these conceptually pleasant and satisfying mechanisms for copying and propagating cell fates, it is less obvious as to how necessary variation of the cell fate is generated. We would like to discuss the possibility whether the dynamic nature of the cell cycle generates the necessary variation in interpreting the same genetic code (Table 1). While the S and M phases of the cell cycle are obligatory and relatively constant in their lengths, there could be significant variation in the length of the gap phases, particularly the gap 1 phase (G1), which represent the major periods of biosynthesis and growth and coincide with the reorganization of chromatin necessary for mitosis [7]. While cell fate specification during normal development appears gradual, cell fate could indeed undergo drastic changes under specific experimental conditions, which highlights the possibility and feasibility that, if the rules that govern cell fate determinations are sufficiently understood, cell fate could be controlled, designed, and engineered for therapeutic purposes.

**Table 1:** Key features of the mammalian cell cycle that could be exploited to alter cell fate

	Conventional mammalian cell cycle (hours) [7]	Ultrafast cell cycle (hours) [8••]	Key molecular events	Events relevant for cell fate determination
G1	11	~1	Cell growth (increase in size) Restriction point active	Reorganizing chromatin after mitosis leading to potential changes in effective concentration and/or compartmentalization of transcription factors [9•, 10••, 11•]
S	8	6	Genome replication	Replicating epigenetic information such as DNA methylation and histone patterns [2–5, 12]
G2	4	~0.5	Cell growth (increase in size) Mitotic checkpoint active	Preparing chromatin for mitosis leading to potential changes in effective concentration and/or compartmentalization of transcription factors
M	1	~0.5	Cytokinesis	Cessation of transcription while key transcription factors remain bound to chromatin to mark active transcription sites in the next cycle [6]

The dramatic cell fate change seen with Yamanaka reprogramming is accompanied with a greatly accelerated cell cycle

## The Extreme Case of Cell Fate Alteration: Reprogramming Somatic Cells Into Pluripotency

Multipotency is one of the defining features of immature stem cells or progenitors, capable of producing a variety of different cell fates. Upon further differentiation, their potency becomes restricted. The specified cells are usually not expected to give rise to cell fates that are significantly different from themselves. However, the cell fate restriction associated with terminally differentiated cells was overturned by the landmark discovery of Shinya Yamanaka and colleagues [13, 14]. They and others have demonstrated that expression of defined factors such as Oct4, Sox2, Klf4, c-Myc (OSKM or the Yamanaka factors) can convert fully differentiated cells into a pluripotent state [13–17]. The success in reprogramming illustrates that cellular identity can be drastically altered even when cells are no longer multipotent. The basic fabric of cell fate could be redesigned and re-displayed with a rather simple combination of factors.

Even though reprogramming can be initiated from most, if not all, somatic cell types, this process is of low efficiency and considered stochastic [18, 19]. The inherent resistance to drastically altering cell fate in response to ectopic transcription factors should be evolutionarily beneficial, since this provides a measure that specialized biochemical functions (e.g., insulin production or action potential transduction) are maintained and malignant transformation is prevented. Viewed in this light, the low efficiency of Yamanaka reprogramming seems logical. The fact that the Yamanaka factors can indeed reconfigure the epigenome, however, begs the question how they achieve this formidable task. Three of the four Yamanaka factors (OSK) can bind to somatic chromatin as pioneer factors [20], which access chromatin irrespective of its epigenetic status. However, many megabase-scaled chromatin domains appear inaccessible to even the pioneer factors [20, 21]. Yamanaka factors could promote direct chromatin remodeling by recruiting such activities onto the chromatin, but why are they successful only in few cells? Additional mechanisms must exist to account for the uniqueness of the few cells, which has been considered purely random in the conventional stochastic view. Cell fate alteration during reprogramming depends on successful interaction between the Yamanaka factors (*trans*-acting factors) and the somatic chromatin (*cis*-acting elements). Since reprogramming remains inefficient in the secondary reprogramming systems [22, 23] where Yamanaka factor expression could be rendered largely comparable across many cells, is it possible that the few cells that do undergo successful reprogramming engage the factors with a more permissive chromatin state? What mechanism(s) could account for such a chromatin state?

Mathematic modeling of the stochastic behavior is consistent with the existence of one major rate-limiting event, and that the long latency culminates in the probabilistic overturn of this limit [19]. Using a direct live-cell imaging approach [24] in attempt to identify this rate-limiting event, we observed that a specific somatic cell type existing in the bone marrow did not display the usual stochastic reprogramming behavior. Rather, their progeny progressed toward pluripotency rapidly, efficiently and largely synchronously [8••]. One of the key features that we could detect was a highly unusual cell cycle behavior: they underwent an ultrafast cell cycle lasting about 8 h/cycle. Fibroblasts are known to accelerate their cell cycle (by Yamanaka factors) prior to activating their endogenous pluripotency. Because the evidence supporting Yamanaka reprogramming to be overall stochastic is irrefutable [18, 19], we reasoned that the non-stochastic reprogramming behavior represents a phase when the major bottleneck event had been overcome. We termed this cell state the “privileged” cell state to convey the fact these cells are more likely to progress toward pluripotency, but additional parameters have to be met (e.g., E-cadherin upregulation) in order for this probability to be realized [8••]. Given that the observed cell cycle speed deviates significantly from most mammalian cell types (Table 1), we surmise that the unusual cell cycle speed might represent a mechanism for generating a more receptive or permissive chromatin state for Yamanaka factors.

We assess the validity of such a hypothesis by examining whether an unusual cell cycle speed represents a *bona fide* facilitator for pluripotency induction. It is clear that a similar cycling behavior is not present with other reprogramming approaches for initiating pluripotency [25]. Pluripotency can be initiated from somatic cells by two alternative approaches besides the Yamanaka approach, namely somatic cell nuclear transfer (SCNT) into oocytes and cell fusion with a pluripotent partner. The time required for pluripotency activation in these processes differs dramatically. While the Yamanaka process generally requires at least 2–3 weeks, SCNT reprogramming follows after only 1–2 cell divisions [19]. Cell fusion-based reprogramming can even occur without any apparent cell division [26]. These observations suggest that cytokinesis per se is not a common denominator prior to pluripotency induction from the somatic nuclei. However, a specific cell cycle-related behavior, i.e., transiting through DNA synthesis and/or its subsequent halving, does appear to be a general facilitator for initiating pluripotency from the somatic state.

In the case of Yamanaka reprogramming, a significant portion of the latency period coincides with the time of cell cycle acceleration [8••]. Indeed, when cell cycle acceleration is accomplished entirely by somatic mechanisms, activation of endogenous Oct4 occurs after 4–5 divisions upon exposure to Yamanaka factors [8••], a likely underestimate due to the relatively low detection sensitivity by imaging as compared to more conventional assays such as Q-PCR. Genetic perturbations that lead to cell cycle acceleration (loss-of-function for cell cycle inhibitors or gain-of-function for CDKs [19, 27–34]) invariably produce more reprogrammed cells. Cell cycle acceleration achieved through other means similarly promotes reprogramming [8••]. Mechanistically, this phenomenon could result from one of two modes of action by the cell cycle. A fast cycling population could provide a larger number of cells with each cell sharing the same probability of progression toward pluripotency or more cells with sufficient cycling speed which are inherently more likely to reprogram. We tested these two scenarios in the context of p53 knockdown and our data were consistent with the latter [8••]. Since DNA replication is obligatory for cell division (with the exception of meiosis), proficient DNA synthesis is a requisite property of the fast cycling cells.

For fusion-based reprogramming, the reprogramming capacity is a function of the cell cycle phase of the pluripotent partner, with S/G2 embryonic stem cells (ESCs) being more potent in reprogramming their somatic partners [35]. Although a potential confounding factor is that cells in the S/G2 phase contain higher gene dosages and could thus be more dominant [36], additional studies support the critical determinant to be cell cycle-related biochemical activities. Specifically, c-Myc promotes DNA replication-dependent reprogramming of the somatic nuclei [37]. Furthermore, fusion of the cytoplasmic materials does not necessarily need to involve two intact cells, as cell-free extracts prepared from mouse pluripotent cells or *Xenopus* eggs could promote pluripotency induction when exposed to somatic cells by transient permeabilization [38, 39]. Strikingly, the promoting effect is restricted to extracts made from M phase cells [38], when DNA content is doubled followed by imminent halving of the genome.

The relevance of cell cycle in SCNT-based reprogramming has been well documented and reviewed elsewhere [40, 41]. On one hand, the success of reprogramming is related to the cell cycle synchrony between the donor cell and the recipient embryonic cell. On the other, the ability of the embryonic cytoplasm to support reprogramming fluctuates according to its cell cycle [42]. While the cytoplasm of interphase zygotes is incapable of reprogramming nuclei from cells beyond the 8-cell stage embryos, the cytoplasm of mitotic zygotes can reprogram adult somatic nuclei [42]. The superiority in reprogramming is not restricted to the cytoplasm provided by the recipient cells, but also could come from the donor somatic chromatin. Specifically, mitotic chromatin are more responsive to the reprogramming activity when transferred into *Xenopus* oocytes, a phenomenon termed “mitotic advantage” [43]. The biochemical property enabling the mitotic advantage appears to be related to ubiquitination-dependent processes [43].

Taken together, although the time duration required for the three main approaches for somatic cell reprogramming seemingly differ from each other, transiting through DNA synthesis appears to universally favor the induction of pluripotency from somatic nuclei. It remains to be determined whether and how chromatin state responds to cell cycle dynamics. Some potential mechanisms leading to an altered chromatin state is discussed below. Cell cycle-based mechanism is likely not the only mechanism for achieving a permissive chromatin state, as transient expression of C/EBPα in B cell progenitors [44] or inhibition of MBD3 [45] have been shown to greatly facilitate pluripotency induction from somatic cells. It does, however, illustrate how the major limitation on cell fate alteration could be overcome in the absence of any additional perturbations other than the Yamanaka factors themselves.

## The Link Between Cell Fate and Cell Cycle: the Exception or the Rule?

The evidence from somatic cell reprogramming systems suggests that both *cis*-acting and *trans*-acting mechanisms could become more competent for cell fate alteration as a consequence of cell cycle dynamics. Both specific cell cycle *phases* and cell cycle *speed* could be involved in achieving this effect. Below, we assess the link between cell cycle and fate determination in a broader context with particular emphasis on discussing cell fate determination during the differentiation of pluripotent stem cells and hematopoietic stem and progenitors.

### 1. Pluripotent Stem Cells.

One of the salient features of ESCs is their rapid cell cycle as compared to somatic cell types. While the doubling time of murine ESCs is about 11 h, the doubling time of somatic cells, as represented by murine embryonic fibroblasts (MEFs), is about 25 h [46, 47]. This rapid cell cycle is primarily due to a severely truncated G1-phase. Human pluripotent stem cells also cycle fast, albeit slower than mouse ESCs [46, 47]. While this difference could be related to the difference between species, another key distinction is the development stage they represent, with human ESCs representing the more differentiated epiblasts [48–50]. Although epiblast stem cells (EpiSCs) are pluripotent and do undergo self-renewal, their pluripotent capacity is more limited compared to ESCs, which is accompanied by an increased cell cycle length, even when compared within the same species [51]. Transitioning between different pluripotency states is associated with changes in the length of cell cycle [52]. Regulators of pluripotency (e.g., Nanog, Oct4, microRNAs) and cell cycle proteins often directly regulate each other while disruption of the pluripotent cell cycle program often compromises pluripotency and leads to differentiation [53–55]. Thus, the maintenance of the pluripotent state requires specific cell cycle dynamics.

On the other hand, differentiation occurs more readily from G1 phase of the cell cycle. Almost 30 years ago, it was shown that differentiation can be induced in G1-phase isolated pluripotent embryonal carcinoma cells (ECCs) by treating with retinoic acid (RA), while ECCs isolated from other cell cycle phases remained impermeable to RA-stimulated differentiation [56]. Recently, G1-phase human ESCs isolated by centrifugal elutriation or by FACS sorting using the FUCCI reporters [57], were shown to be especially amenable to differentiation [9•, 12, 58]. These data reveal that commitment to differentiation is preferentially initiated in or limited to G1-phase. Mechanistically, Pauklin and Vallier demonstrated that Smad2/3-mediated activation of endoderm-specific genes in human ESCs only occurs in early G1, when cyclin D/Cdk4/6 activity is low and ineffective in limiting the nuclear localization of Smad2/3 [9•]. Additionally, the deposition of epigenetic marks is regulated by cell cycle [12].

The canonical G1-phase has two sub-phases, an early G1 before the restriction point (R point) and late G1. The R point is the turning point at which the cell commits to another round of DNA replication. Overcoming the R point occurs through inhibitory phosphorylation of retinoblastoma protein (Rb), resulting in the liberation of E2F, thereby activating E2F-mediated transcription of S-phase genes. Early G1-phase is highly susceptible to regulatory signals. After the R point is passed, the cell is no longer sensitive to extracellular signals and commits toward S-phase progression. ESCs have a nonexistent early G1, characterized by the absence of hypo-phosphorylated Rb [59]. This cell cycle structure is consistent with the fact that pluripotency can be propagated in the absence of serum and growth factors [60], indicating that early G1 shortening might be particularly important for pluripotency. Since Cdk2 activity mediates G1/S transition in late G1 phase, inhibition of Cdk2 activity might not necessarily compromise the early G1 phase truncation in ESCs, which may account for the inefficiency of Cdk2-inhibitors in perturbing pluripotency [61]. Alternatively, the length (or a threshold of length) of G1 phase might be less critical when the cell identity only needs to be copied (i.e., self-renew) but becomes more relevant upon exiting from pluripotency, as longer G1 may allow lineage-specific transcription factors to bind their target sites after being displaced from condensing chromatin during mitosis [41].

### 2. Multipotent Hematopoietic Stem and Progenitors.

The hematopoietic system is one of the best charted adult developmental programs regarding cell fate specification and cell cycle properties. On the top of the hematopoietic hierarchy, hematopoietic stem cells (HSCs) sustain life-long blood production, a function that is dependent upon its extensive quiescence. The majority of HSCs reside in G0 [62] with some entering cell cycle once every ~145 days [63]. HSCs can switch between quiescence and cycling depending on signals of tissue injury or inflammation [63, 64]. Specifically, the long-term repopulating potential predominantly resides in the quiescent pool, particularly in the G0 fraction [62, 65]. HSC quiescence is controlled by both intrinsic genetic programs and extrinsic factors from the bone marrow niche [66]. Interfering with cell cycle machinery in HSCs usually impacts HSC behavior [66]. Conversely, perturbations that alter HSC fate outcome are often reflected as a change in their cycling behavior [67, 68]. Physiological aging is associated with HSC functional decline concomitant with increased HSC proliferation [66, 69]. Although the reduction in HSC function could be a consequence of replicative exhaustion, it is likely that this change is a direct result of cell cycle entry, given that exposure to cytokines or growth factors stimulate HSCs to enter the cell cycle while reducing their long-term engraftment capabilities [70, 71].

A direct example of cell cycle influencing HSC fate outcome is seen at one of the earliest cell fate bifurcation points. While retaining potency toward both myeloid and lymphoid lineages, HSCs display inherent lineage bias, with some producing more lymphoid progeny while others more myeloid [72]. Importantly, lymphoid-biased HSCs are more proliferative than myeloid-biased HSCs [73]. The mechanism of lymphoid versus myeloid fate choice imparted by varying the cell cycle kinetics is elegantly depicted with the transcription factor PU.1 [10••], whose steady state concentration is determined by cell cycle length coupled with its protein stability. In slower cycling cells, PU.1 accumulates which drives a myeloid fate [10••]. While this was demonstrated specifically for PU.1, it is conceivable that similar mechanisms may contribute to the effectiveness of other key fate specifying transcription factors, with their steady state concentration being a function of the multipotent progenitor cell cycle length as well as their inherent messenger RNA (mRNA) and/or protein stability.

### 3. Other lineages.

The intimate relationship between cell cycle and cell fate control is not unique to pluripotent stem cells and HSCs. Within the hematopoietic lineage, a much later fate specification event is accompanied by distinctive cell cycle regulation, perturbation of which leads to impaired erythropoiesis associated with altered DNA methylation and inadequate mitochondria function [74–76]. Endothelial cell fate specification illustrates another case where cell fate determination is tightly coupled to cell cycle. During early development, endothelial cells arise from mesoderm shortly after gastrulation and differentiate into arterial, venous, lymphatic, and hemogenic lineages to establish the vascular and hematopoietic systems, under the control of both extrinsic and intrinsic mechanisms [77]. Disruption of vascular endothelial growth factor (VEGF) or RA signaling leads to endothelial cell hyper-proliferation and impaired vascular maturation [78–80]. In the developing cortex, a correlation between G1 lengthening and differentiation has been extensively studied [81–83]. While inhibiting the cell cycle of neural progenitor cells (NPC) promotes neurogenesis, enhancing cell cycle progression leads to NPC self-renewal and decreased neurogenesis. In the adult heart, Rb participates in maintaining the postmitotic state of adult cardiac myocytes by directing heterochromatin formation [84]. Reactivation of cardiac regeneration follows cell cycle re-entry of the cardiomyocytes [85].

Taken together, a relationship between cell fate choice and cell cycle control is general, if not universal, as cell cycle phase and/or speed influences the key fate determination points in multiple germ layers involving many distinctive cell types.

## Potential Mechanisms

As discussed above, cell cycle dynamics could impart a competent cell state facilitating mechanisms acting *in trans* as well as *in cis*. Chromatin change in relation to cell cycle has been reviewed timely and comprehensively by Buttitta and colleagues [86] and will not be covered here. In a simplistic view, the obligatory genome replication accompanying cell cycle progression creates plentiful opportunities and possibilities for variation in copying the entire epigenome (Table 1). Cell fate is synonymous for different epigenetic configuration relying on DNA methylation, histone tail modification, histone variants deposition, chromatin association with the nuclear lamina as well as their organization in the 3D space. Watson-Crick base pairing and mitotic bookmarking [6] only provide the means of copying the DNA sequence itself and some of the basic gene expression “memory,” respectively. It will be important to define the specific mechanisms of how individual components of the epigenome are laid down during the cell cycle. In addition to varied epigenome copying, cell cycle dynamics could effectively generate a gradient of key transcription factor concentrations [10••] or provide alternating compartmentalization between the interaction of *trans*- and *cis*- elements [9•, 11•]. On the other hand, we note the absence of apparent cell cycle involvement in trans-differentiation of other lineages [87–90]. It is possible that the conversion of closely related lineages is not as severely restricted by the differences in their epigenome, such as during C/EBPα-mediated B cell to macrophage conversion and Pdx1/Ngn3/MafA-mediated pancreatic exocrine to endocrine cell conversion [89, 90]. In these cell fate conversions, providing appropriate transcription factors might suffice. Furthermore, lack of apparent cytokinesis does not necessarily negate the involvement of cell cycle-related mechanisms, such as in the case of endomitosis during megakaryocyte differentiation [91].

In addition to the above-mentioned mechanisms, passive DNA demethylation represents a powerful cell division-coupled mechanism to reset the epigenome, which is active during pre-implantation embryonic development [92, 93] as well as primordial germ cell (PGC) specification [94, 95]. Both processes are accompanied by a period of rapid cell cycling at a speed of 8–12 h per cycle. Mammalian DNA methylation on cytosine is catalyzed by the DNA methyltransferases (Dnmts). Mammalian cells express one maintenance DNA methyltransferase, Dnmt1, which displays high binding affinity to semi-methylated DNA. It is recruited to the replication fork during S phase, where it binds to and methylates the newly synthesized DNA strand (CpG di-nucleotide) using the mother strand methylation pattern as a template [96]. Chemical or genetic inhibition of Dnmt1 in proliferating cells could lead to passive loss of DNA methylation. In the case of PGC specification, Dnmt1 recruitment to the replication forks appears to be compromised [94]. It remains to be determined whether passive DNA demethylation is functionally relevant during additional cell fate specification processes. Supporting such a possibility is the recent report that a specific epigenetic mark, 5-hydroxymethylcytosine (5hmC) level oscillates during the ESC cycle with its level peaking at late G1 and declining in S phase [12].

Besides its involvement in SCNT reprogramming, cell cycle synchrony among early embryonic cells predicts successful development in both human and mice [97, 98]. Highly predictive parameters for successful embryonic development include the duration of the first cytokinesis, the time interval between the first and the second mitoses, and the time interval between the second and third mitoses [98]. These parameters are exploited in designing the EmbryoScope to identify good-quality embryos used in in vitro fertilization (IVF) clinics. Similarly, reduced synchrony is associated with aberrant embryonic genome activation and perturbed metabolic reprogramming during early mouse embryonic development [97]. It remains to be determined whether the cell cycle abnormality is the cause or consequence of the failing development and how a rather mild difference in cell cycle synchrony is translated into aborting development all together. Nonetheless, successful development depends on precise cell cycle control.

## Current Challenges: Reporting the Cell Cycle Speed

From the discussion above, it becomes obvious that the current tools for analyzing cell cycle are inadequate. Currently available tools mostly focus on specific cell cycle phases, while approaches for direct and quantitative measurement of cell cycle speed are rather limited. Experimental approaches for inducing synchrony [99] greatly enriches for cells in specific phases, but it also introduces significant interference with the natural physiology.

Conventional cell cycle assays usually exploit the unique biochemical or biophysical properties associated with cell cycle progression. Many of these assays are based on increased DNA content/synthesis by staining DNA itself or the replication machinery components such as PCNA or Ki67 [63]. Cell cycle progression is propelled by oscillating cyclins, their associated CDKs and their targets, which underlies the design of several cell cycle phase reporters such as the cyclin B1-GFP [100] and FUCCI reporters [57]. Physical properties could also be used. For example, mitotic shake-off enriches for cells in M phase among adherent cells based on the fact that mitotic cells display rounded morphology and temporarily reduced adhesion [99]. Another feature of cycling cells is that their size is smaller right after mitosis, a principle used in centrifugal elutriation [101]. While mitotic shake-off and elutriation are free of label or genetic manipulation, they also face significant limitations. For example, mitotic shake-off would not be applicable for floating cells or cells from fresh tissues. Cell size is not always indicative of cell cycle status, e.g., quiescent long-term HSCs are much smaller than their actively cycling downstream progenitors.

Dye dilution and live-cell imaging are two main approaches for resolving cell cycle speed and/or synchrony. A common way of identifying quiescent adult stem cells is the “label retention” approach. In these experiments, populations of heterogeneous and asynchronous cells are exposed to a defined period of labeling (i.e., “pulse” by BrdU, GFP-tagged histones, or CellTrace dyes) and followed by another period of label dilution [63, 64]. Quiescent stem cells are enriched among the label-retaining cells. The major drawback of the dye-labeling approach is that a significant amount of proliferation is necessary for resolving the differences in cycling behaviors, by which time the label-retaining cells and label-diluted cells have departed from their original state of analysis. Furthermore, dye dilution only provides a relative measure among a population as to which cells have undergone more cell divisions, but does not provide the absolute measure as to how long each cell cycle lasts. The resolving power is thus severely limited among homogeneous cells. Alternative to dye dilution, live-cell imaging measures the absolute duration of each cell cycle. Combined with the fluorescent cell cycle phase reporters, durations of specific cell cycle phases could be determined [8••]. Although a powerful approach in cell fate analysis, imaging requires at least two cell divisions to derive a measurement for a full cell cycle. Imaging for the duration of several cell cycles usually demands sophisticated capacities in image acquisition, annotation, and tracking, limiting its wide application.

A cell cycle speed reporter, presumably fluorescence-based, would thus greatly benefit cell fate analysis. Toward this goal, Peacock et al. reported the use of an E2F activity reporter [102]. When normalized to the activity of a constitutive mCherry reporter, the E2F reporter gives a twofold fluctuation consistent with cell cycle progression. While this approach demonstrates proof-of-principle, its application requires stable integration of two genetically encoded fluorescence proteins with a limited dynamic range. Alternatively, endogenous cell cycle drivers fused with fluorescent reporters could be considered, such as the GFP-c-Myc fusion protein [103]. Novel designs for reporting the cell cycle speed need to be implemented.

## Implications for Clinical Application

Like HSCs, many other adult stem cells display extensive quiescence. Unlike HSCs, ESCs have a severely truncated G1. Since both ESCs and adult stem cells are capable of extensive self-renewal, or perpetuating their own cell fate, the two scenarios might represent two strategies to avoid frequent loitering in the early G1 phase, when cells respond readily to external stimuli. Viewed in this light, derivation of therapeutic cell types would follow different strategies. Derivation of somatic lineages should benefit from lengthening the G1 phase of pluripotent stem cells. Indeed, lengthening G1 duration by simply exposing human ESCs to dimethyl sulfoxide (DMSO) enhanced differentiation toward all three germ layers tested [104••]. The implication for deriving HSCs, however, is to prolong the G0 phase which is incompatible with the goal of expanding these cells. This might explain the overall inability to effectively expand HSCs in vitro, since most expansion strategies involve activating HSCs into cell cycle by growth factors. The few specific cases in which HSCs could be amplified, such as by overexpressing HOXB4 or miR-125 [105, 106], offer excellent opportunities to investigate how these genetic factors bypass the cell cycle requirement to reinstate the HSC fate.

## Conclusions

As discussed above, the mechanisms of cell fate control are intimately coupled to cell cycle regulation in a wide variety of biological systems. The dynamic nature of the cell division cycle could provide many opportunities to alter the gene expression programs, as a result of altered chromatin configuration or key transcription factor concentration or the interaction between them. Understanding the fundamental rules of cell fate choices is necessary for harnessing the cell fate plasticity for regenerative medicine, which implicates a likely intimate relationship with cell cycle control. Extensive tissue regeneration might not be unique to planarians or newts after all, as the MRL mice which display superior wound healing ability is enabled by a natural deficiency in p21 [107].
